# Current Induced Heat Generation in Ferromagnet-Quantum Dot-Ferromagnet System

**DOI:** 10.3390/ma8073854

**Published:** 2015-06-25

**Authors:** Lili Zhao, Qiao Chen, Yamin Zhang, Lina Zhao

**Affiliations:** 1 Department of Fundamental Courses, Academy of Armored Force Engineering, Beijing 100072, China; E-Mail: zhaolili219@aliyun.com; 2 Department of Maths and Physics, Hunan Institute of Engineering, Xiangtan 411104, Hunan, China; E-Mail: cqhy1127@aliyun.com; 3 Mechanical and electrical engineering college, Yanching Institute of Technology, Langfang 065201, Hebei, China; E-Mail: QQ328157598@aliyun.com; 4 Key Laboratory for Biomedical Effects of Nanomaterials and Nanosafety, Institute of High Energy Physics, Chinese Academy of Sciences, Beijing 100049, China

**Keywords:** heat generation, ferromagnet terminals, non-equilibrium Green’s functions, metamaterials

## Abstract

We study the heat generation in ferromagnet-quantum dot-ferromagnet system by the non-equilibrium Green’s functions method. Heat generation under the influence of ferromagnet leads is very different compared with a system with normal metal leads. The significant effects in heat generation are caused by the polarization angle θ associated with the orientation of polarized magnetic moment of electron in the ferromagnetic terminals. From the study of heat generation *versus* source drain bias (Q-eV) curves, we find that the heat generation decreases as θ increases from 0 to 0.7π. The heat generation *versus* gate voltage (Q-eV_g_) curves also display interesting behavior with increasing polarization angle θ. Meanwhile, heat generation is influenced by the relative angle θ of magnetic moment in the ferromagnetic leads. These results will provide theories to this quantum dot system as a new material of spintronics.

## 1. Introduction

With the development of industry and information technology, various nano-devices have been designed and fabricated in laboratories, such as the electromagnetic quantum dot (QD) constructed single electron diodes and transistors [1,2,3,4]. From a microscopic point of view, the heat generation in electromagnetic nano-devices originates mainly from the inelastic electron-phonon (EP) scatting [5]. Because these electromagnetic nano-devices could be integrated with an extreme high density, their heat generation and thermal dissipation have become urgent problems. Until now, an increasing number of theoretical and experimental works have focused on the heat generation problem in nanoscale structures [6,7,8,9]. Sun and Xie proposed a general formula for heat generation in lead-QD-lead system by non-equilibrium Green’s functions (NEGF) method. They found that the behaviors of heat generation in this case are quite different from those in the usual macroscopic one and that the Joule law no longer holds true [10,11]. The single molecular nano-junction also presents the heat generation, which is unique to nanostructures, revealing significant difference from heat generation in macroscopic systems by using first-principles approaches [12]. Experimentally, Huang *et al.* demonstrated current-induced local heating effects in a single molecule [13,14]. In addition, thermoelectric transport has also attracted great interest from physicists [15,16,17,18,19].

Besides the electric nano-devices, spintronic devices employ the concept of controlling the spin degree of freedom in addition of charge. To construct spintronic devices, one should generate the spin polarized electrons to tunnel through a definite device. Ferromagnetic lead-based spintronic devices have been widely researched in recent years [20,21,22,23]. Using ferromagnetic electrodes, the scattering region becomes spin polarized by the local exchange field and we can construct spin filters, spin transistors and spin memories. The spin-valve effect also exists in ferromagnetic leads coupled systems. The motivation for employing the specific effects of spin in materials naturally enables scientists to contrive novel spintronic nano-devices for magnetoelectronical applications [24,25,26,27]. The heat generation problem is proposed in the spintronic nano-device with tunneling electrodes (at least one ferromagnetic lead) at different temperatures [24]. However, this is far from clear for the heat generation characteristics of spintronic lead coupling devices. These effects could cause the novel behaviors of heat generation which are important in controlling the heat generation performance of spintronic nano-devices. In this work, we study the heat generation in ferromagnet-QD-ferromagnet system by non-equilibrium Green’s functions method. The significant effects in heat generation are caused by the polarization angle associated with the orientation of a polarized magnetic moment of the electron in the ferromagnetic terminals. The paper is organized as follows. In Section 2, we present the Hamiltonian of the coupling system and detailed algebraic expressions by the NEGF method. The matrix form of the current and heat generation formulas are also given there. The numerical results and analyses are performed in Section 3. The final section is arranged for conclusion remarks.

## 2. Model and Formalism

**Figure 1:**
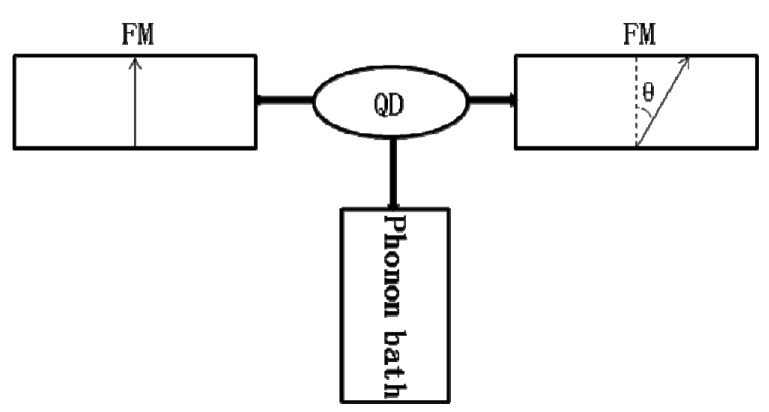
Schematic diagram of a quantum dot (QD) coupled to two ferromagnetic leads and phonon reservoirs. The magnetizations between the left and the right ferromagnetic (FM) leads deviate by angle θ.

We build the spin nano-device prototype as ferromagnet-QD-ferromagnet (FM-QD-FM) structure shown in Figure 1. The system consists of one QD coupled to the left and right ferromagnetic terminals, which could present a general heat generation expression for its mesoscopic transport. The magnetic moment *M*_γ_ of the γ-th electrode is tilted at angle θ_γ_ to the ***e****_z_* direction, where ***e****_z_* is the unit vector perpendicular to the direction of tunneling current ***e****_x_*. The Hamiltonian of the coupling system can be written as the universal expression [28,29,30]:

(1) $$\begin{array}{l} H=\sum_{\gamma \kappa \sigma }\{\left[\varepsilon_{\gamma \kappa }\left(t\right)-\sigma M_{\gamma }\cos\theta_{\gamma }\right]\alpha_{\gamma \kappa \sigma }^{+}\alpha_{\gamma \kappa \sigma }-M_{\gamma }\sin\theta_{\gamma }\alpha_{\gamma \kappa \sigma }^{+}\alpha_{\gamma \kappa \bar{\sigma }}\}+\\ \;\sum_{\sigma }\left[\varepsilon_{d}+\lambda_{q}(\alpha_{q}^{+}+\alpha_{q})\right]d_{\sigma }^{+}d_{\sigma }+\sum_{\gamma \kappa \sigma }\left(T_{\gamma \kappa }\alpha_{\gamma \kappa \sigma }^{+}d_{\sigma }+H.c.\right)+\sum_{q}\omega_{q}\alpha_{q}^{+}\alpha_{q} \end{array}$$

where γ ∊ {*L*, *R*}. *H* is the Hamiltonian of the coupling system. In the Hamiltonian, α^+^_γκσ_ and α_γκσ_ represent the creation and annihilation electron operators of the γ-th ferromagnetic terminals with momentum κ and spin σ. d^+^_σ_ and d_σ_ are the creation and annihilation electron operators of the QD. ε_γκ_ are the isolated energies of terminals and ε_d_ is the isolated energy of QD. The magnitude *M*_γ_ = ½*g*μ_B_*h*_γ_ is associated with the Bohr magneton μ_B_, Landé factor *g*, and the molecular field strength *h*_γ_ of the γ-th terminal. α^+^_q_ and α_q_ are the creation and annihilation operators of a phonon having frequency ω_q_. The quantity λ_q_ is the electron-phonon coupling strength. *T*_γκ_ is the coupling strength of central QD with the γ-th ferromagnetic terminal. *H.c.* means hermitian conjugation.

We further make the transformation over the Hamiltonian by introducing the creation and annihilation operators of quasi-particles α^+^_γκσ_ and α_γκσ_ *via* the unitary transformation $\alpha_{\gamma \kappa \sigma }=\alpha_{\gamma \kappa \sigma }cos\frac{\theta_{\gamma }}{2}-\sigma \alpha_{\gamma \kappa \bar{\sigma }}sin\frac{\theta_{\gamma }}{2}$. $\bar{\sigma }$ is also spin which takes the opposite value with σ. This procedure transforms the spin-flip effect from the ferromagnetic terminals to the tunneling terms. After this transformation, the Hamiltonian of the left lead is diagonalized as:

(2) $$H=\sum_{\text{γκ}\sigma }\left[{\text{ε}}_{\text{γκ}}(t)-\sigma M_{\text{γ}}\cos\theta_{\text{γ}}\right]\alpha_{\text{γκσ}}^{+}\alpha_{\text{γκσ}}$$

The interaction terms between the ferromagnetic leads and the QD are now written as the elements ${\tilde{T}}_{\gamma \kappa \sigma \sigma^{\prime }}$ of the interaction strength matrix ${\tilde{T}}_{\gamma \kappa }={\tilde{T}}_{\gamma \kappa }R\left(\theta_{\gamma }\right)$ in the spin-space, where the rotation matrix is characterized by the following matrix with its off-diagonal elements describing the spin-flip effect:

(3) $$R\left(\theta_{\gamma }\right)=\left(\begin{array}{cc} cos\theta_{\gamma }/2 & sin\theta_{\gamma }/2\\ -sin\theta_{\gamma }/2 & cos\theta_{\gamma }/2 \end{array}\right)$$

So the total Hamiltonian of the system after the transformation:

(4) $$\begin{array}{l} H=\sum_{\text{γκσ}}\left[{\text{ε}}_{\text{γκ}}(t)-\sigma M_{\text{γ}}\cos\theta_{\text{γ}}\right]\alpha_{\text{γκ}\sigma }^{\text{+}}\alpha_{\text{γκ}\sigma }+\sum_{\sigma }\left[{\text{ε}}_{\text{d}}+{\text{λ}}_{\text{q}}(\alpha_{\text{q}}^{+}+\alpha_{\text{q}})\right]d_{\sigma }^{+}d_{\sigma }\\ +\sum_{\text{γκ}\sigma \sigma^{'}}\left(\alpha_{\text{γκ}\sigma }^{\text{+}}{\tilde{T}}_{\text{γκ}\sigma \sigma^{\text{'}}}d_{\sigma^{\text{'}}}+H.c.\right)+\sum_{\text{q}}{\text{ω}}_{\text{q}}\alpha_{\text{q}}^{+}\alpha_{\text{q}} \end{array}$$

The heat generation in the QD per unit time at time *t* can be calculated from the time evolution of the operator $E_{ph}=\sum_{q}\omega_{q}\alpha_{q}^{+}\alpha_{q}$:

(5) $$Q(t)=\langle dE_{ph}/dt\rangle$$

*E*_ph_ is the energy of phonon.

By equation of the motion (EOM) method, we obtain:

(6) $$Q(t)=-2Re\sum_{q\sigma }\lambda_{q}\omega_{q}i\langle \alpha_{q}^{+}d_{\sigma }^{+}d_{\sigma }\rangle$$

Q(t) is the heat generation, *Re* means the real part of the formular.

We define Green’s functions [9,10]:

(7) $$G_{q\sigma }^{<}(t,t^{\prime })=-i\langle \alpha_{q}^{+}\left(t^{\prime }\right)d_{\sigma }^{+}\left(t\right)d_{\sigma }\left(t\right)\rangle$$

(8) $$G_{q\sigma }^{r}(t,t^{\prime })=-i\theta \left(t-t^{\prime }\right)\langle \left[d_{\sigma }^{+}\left(t\right)d_{\sigma }\left(t\right),\alpha_{q}^{+}\left(t^{\prime }\right)\right]\rangle$$

$G_{q\sigma }^{<}(t,t^{\prime })$ is Keldysh Green’s function, $G_{q\sigma }^{r}(t,t^{\prime })$ is the retarded Green’s function.

Using these Green’s functions, we rewrite the heat generation in Equation (6) as follows:

(9) $$Q\left(t\right)=2Re\sum_{q\sigma }\lambda_{q}\omega_{q}G_{q\sigma }^{<}(t,t)$$

In order to obtain the formulas for electronic current and heat current through FM-QD-FM system, we follow the same way described in reference [31,32,33] and express the electronic current and the heat generation with Keldysh Green’s functions. Then, we can obtain the formulas for the heat generation:

(10) $$Q=Re\sum_{\sigma \sigma^{\prime }}\omega_{q}\lambda_{q}^{2}\int \frac{d\omega }{2\pi }\left\{{\tilde{G}}_{\sigma \sigma^{\prime }}^{<}(\omega ){\tilde{G}}_{\sigma^{\prime }\sigma }^{>}(\tilde{\omega })-2N_{q}\left[{\tilde{G}}_{\sigma \sigma^{\prime }}^{<}(\omega ){\tilde{G}}_{\sigma^{\prime }\sigma }^{\alpha }(\tilde{\omega })+{\tilde{G}}_{\sigma \sigma^{\prime }}^{r}(\omega ){\tilde{G}}_{\sigma^{\prime }\sigma }^{<}(\tilde{\omega })\right]\right\}$$

where $\tilde{\omega }=\omega -\omega_{q}$. *N*_q_ is the phonon population and can be expressed as *N*_q_ = 1/[exp(ω_q_/*k*_B_*T*_ph_) − 1]. *T*_ph_ is the temperature of the phonon bath.

The retarded Green’s function has been obtained in the studies of transport:

(11) $${\tilde{G}}_{\sigma \sigma^{\prime }}^{r}(\omega )=\frac{\zeta_{\bar{\sigma }\bar{\sigma }}\left(\omega \right)\delta_{\sigma \sigma^{\prime }}+\Sigma_{\sigma \bar{\sigma }}^{r}\left(\omega \right)\delta_{\bar{\sigma }\sigma^{\prime }}}{\zeta_{\sigma \sigma }\left(\omega \right)\zeta_{\bar{\sigma }\bar{\sigma }}\left(\omega \right)-\Sigma_{\sigma \bar{\sigma }}^{r}\left(\omega \right)\Sigma_{\bar{\sigma }\sigma }^{r}\left(\omega \right)}$$

where $\zeta_{\sigma \sigma^{\prime }}\left(\omega \right)=\varepsilon -\varepsilon_{d}-\Sigma_{\sigma \sigma^{\prime }}^{r}\left(\omega \right)$.The self-energy matrix $\Sigma_{\sigma \sigma^{\prime }}^{r}\left(\omega \right)$ represents the total self-energy matrix of the terminals $\Sigma_{\sigma \sigma^{\prime }}^{r}\left(\omega \right)=\sum_{r}\Sigma_{\sigma \sigma^{\prime }}^{r}\left(\omega \right)$. The Keldysh Green’s functions are given by ${\tilde{G}}_{\sigma \sigma^{\prime }}^{<,>}(\omega )={\tilde{G}}_{\sigma \sigma^{\prime }}^{r}(\omega ){\tilde{\Sigma }}_{\sigma \sigma^{\prime }}^{<,>}(\omega ){\tilde{G}}_{\sigma \sigma^{\prime }}^{\alpha }(\omega )$, where the self-energy functions are expressed as ${\tilde{\Sigma }}^{<,>}(\omega )=\sum_{r}{\tilde{\Sigma }}_{r}^{<,>}(\omega )$, ${\tilde{\Sigma }}_{r}^{<}(\omega )=i{\tilde{\Gamma }}_{r}f_{r}(\omega )$ and ${\tilde{\Sigma }}_{r}^{>}(\omega )=-i{\tilde{\Gamma }}_{r}\left(1-f_{r}(\omega )\right)$. ${\tilde{\Sigma }}_{\sigma \sigma^{\prime }}^{<,>}(\omega )$ are the lesser and greater self-energies.

The elements of the matrix ${\tilde{\Gamma }}_{\gamma }$ is determined by the following functions ${\tilde{\Gamma }}_{\gamma \sigma \sigma }=cos^{2}\frac{\theta_{\gamma }}{2}{\tilde{\Gamma }}_{\gamma \sigma }+sin^{2}\frac{\theta_{\gamma }}{2}{\tilde{\Gamma }}_{\gamma \bar{\sigma }}$:

(12) $${\tilde{\Gamma }}_{\gamma \sigma \bar{\sigma }}=\sigma cos\frac{\theta_{\gamma }}{2}sin\frac{\theta_{\gamma }}{2}\left({\tilde{\Gamma }}_{\gamma \sigma }-{\tilde{\Gamma }}_{\gamma \bar{\sigma }}\right)$$

${\tilde{\Gamma }}_{\gamma \sigma }$ is the modified linewidth function given by ${\tilde{\Gamma }}_{\gamma \sigma }=\Gamma_{\gamma \sigma }exp\left[-{\left(\lambda_{\theta }/\omega_{\theta }\right)}^{2}\left(2N_{q}+1\right)\right]$. The current formula:

(13) $$I_{L}=\frac{e}{h}\sum_{n}Tr\int d\omega L_{n}{\tilde{\Gamma }}_{L}{\tilde{G}}^{r}\left(\omega -n\omega_{\theta }\right){\tilde{\Gamma }}_{R}{\tilde{G}}^{\alpha }\left(\omega -n\omega_{\theta }\right)\left[f_{L}\left(\omega \right)-f_{R}\left(\omega \right)\right]$$

where $L_{n}=e^{-g\left(2N_{ph}+1\right)}I_{n}\left(2g\sqrt{N_{ph}\left(N_{ph}+1\right)}\right)e^{n\omega_{q}\beta /2}$, $\beta =1/\left(k_{B}T_{ph}\right)$, *I_n_* is the *n*-th Bessel function of the complex argument. *Tr* means the trace of the matrix.

At zero temperature, as *n* ≥ 0:

(14) $$L_{n}=e^{-g}g^{n}/n!$$

As *n* < 0, *L_n_* = 0.

## 3. The Numerical Calculations

In this section, we perform numerical calculations to examine and control the heat generation in FM-QD-FM system. There is no Coulomb interaction in the central QD. We only consider a single level QD system by setting ε_d_ = 0 to study the behavior of heat generation around a single level. We take Δ = 1.0 meV as the energy scale in the numerical calculation, which indicates all of the energy quantities are compared to it. The linewidths are considered to be energy independent in the wide-band limit, but to be spin-dependent. The parameters are chosen as Γ_L↑_ = Γ_R↑_ = 0.3Δ, Γ_L↓_ = Γ_R↓_ = 0.09Δ, λ = 1.0Δ, ω_q_ = 1.0Δ. We set the polarization angle of the left terminal to be zero θ_L_ = 0, and examine the heat generation variation at arbitrary polarization angle of the right terminal by denoting θ_R_ = θ. The same temperature of phonon and electron, *T*_q_ = *T*_e_ = *T* are considered throughout the numerical calculations. The chemical potential of the right lead is chosen as the energy zero point μ_R_ = 0, and then the bias voltage is eV = μ_L_. We calculate the heat generation *versus* the source-drain bias, gate voltage with different polarization angle θ. The phonon energies ω_q_ and λ^2^_q_ are taken as the scale of energy and heat generation, respectively.

**Figure 2:**
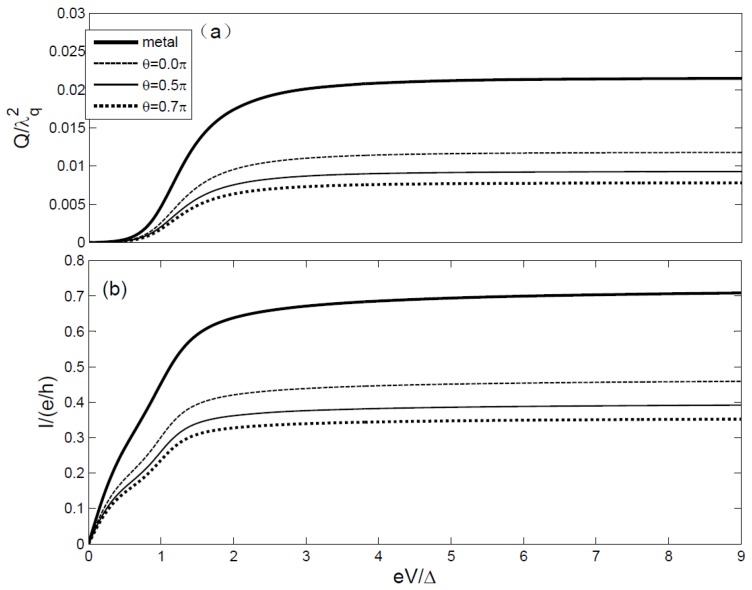
(**a**)The heat generation *Q versus* the source-drain bias eV with different polarization angles θ. (**b**) The current *I versus* the source-drain bias eV with different polarization angles θ. The parameters are chosen as the gate voltage eV_g_ = 0, K_B_T = 0.1.

Figure 2a,b depict the heat generation *Q* and the current *I versus* source-drain bias eV with different polarization angles θ respectively. One can observe that the heat generation decreases with the increasing spin polarization angle in Figure 2a. As the angle between the magnetizations increases, the charge current is reduced and at the same time so is the heat generation due to the spin-valve effect. Both the electric current and the heat generation increase with increasing bias voltage. However, the rapid jumps of the current and the heat generation happen at different bias voltage. The increase of the heat generation has a delay of ω_q_ with respect to the electric current. The reason for this is that the heat generation originates from the process of phonon emitting when an electron is at the state of ω jumping to an empty state of ω − ω_q_. One can see that many small sub-steps emerge in the current curves because of the phonon-assisted tunneling processes, but no phonon assisted sub-steps in the heat generation curves. This reason for this is that they are obtained respectively in the electron and electron-phonon interaction representation. The current and the heat generation in FM-QD-FM system are deeply suppressed compared with the metal-QD-metal system.

**Figure 3:**
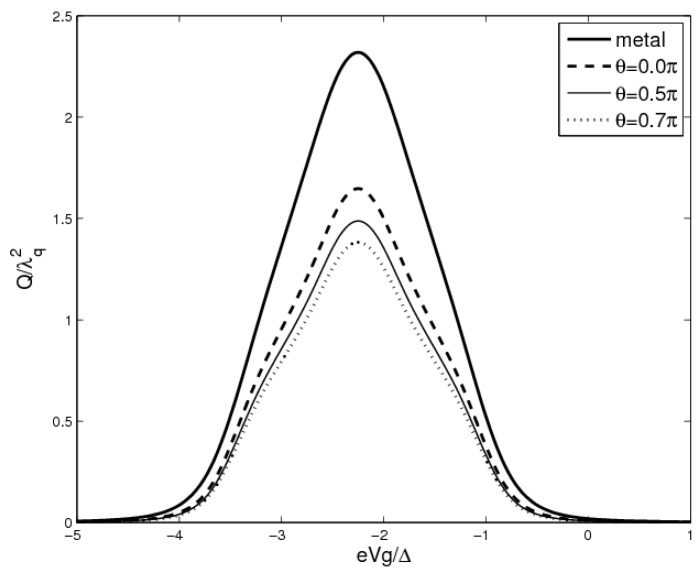
The heat generation *Q* *versus* the gate voltage eV_g_. The parameters are chosen as eV = 2.5Δ, K_B_T = 0.1.

In Figure 3, we study the heat generation *Q versus* gate voltage without Coulomb interaction. The heat generation in metal-QD-metal system appears one resonant peak with its magnitude being about 2.4λ^2^*_q_*. In contrast, the heat generation in FM-QD-FM system is deeply suppressed due to the spin-valve effect. The heat generation always shows symmetric peak in the absence of magnetic fields and Coulomb interaction [10]. Different curves are plotted for different polarization angles, and the magnitude of heat generation is intimately related to the polarization angle in FM-QD-FM system. In fact, the heat generation decreases as θ increases from 0 to 0.7π shown in diagram. Therefore, we could control the heat generation performance by adjusting the polarization angles in ferromagnetic leads.

**Figure 4:**
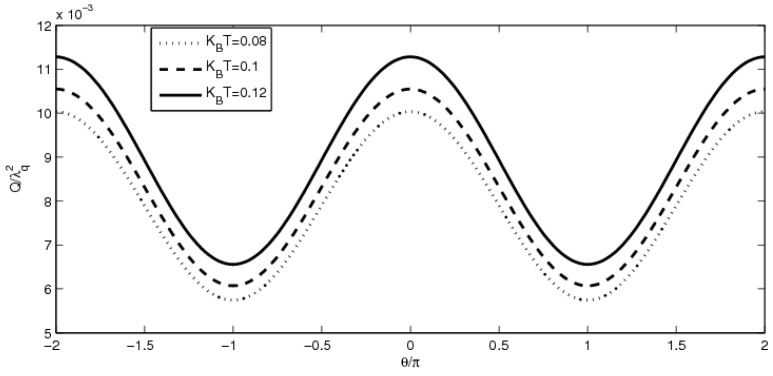
The heat generation *Q* *versus* polarization angle θ. The parameters are chosen as eV = 2.5Δ, eV_g_ = 0.

In order to undertake further investigation into the spin polarization originated from the ferromagnetic terminals, we present the heat generation *Q* *versus* the polarization angle θ at different magnitudes of temperature in Figure 4. It is clear to find that the heat generation varies with polarization angle periodically with the period 2π. Heat generation increases with increasing magnitudes of temperature. The valley of the heat generation at θ = 0 rises, while the two valleys at θ = ±π become deeper to form wider heat plateau as the temperature is higher enough. This means that the suppression and enhancement of heat generation for different absorption and emission cases are sensitively dependent on the magnitudes of temperature and the polarization angle.

The spin-flip effect will occur under the perturbation of magnetic fields, which could bring new physical phenomena. The coupling between spin-up and spin-down components causes novel transport in the nonmagnetic material system. The spin-flip effect may also provide some theoretical support to make electromagnetic metamaterials. The QD-based spintronic devices have attractive advantages, such as faster data-processing speed, and less electric power consumption. The heat in a solid-state device originates mainly from the inelastic electron-electron and electron-phonon scattering; we can decrease the heat generation by increasing the polarization angle θ. The transport properties in quantum dot attached ferromagnetic leads can be controlled with the aid of the electron spin degree of freedom.

## 4. Conclusions

We have investigated the heat generation in FM-QD-FM system by NEGF method. The spin polarized electrons play an important role in heat generation. The polarization angle is associated with the orientation of electronic polarized magnetic moment in the ferromagnetic terminals. It contributes significantly to heat generation. There are many small sub-steps emerging in the current curves because of the phonon-assisted tunneling processes, but no phonon assisted sub-steps in the heat generation curves.

The current and the heat generation in FM-QD-FM system are deeply suppressed compared with the metal-QD-metal system. The heat generation decreases as θ increases from 0 to 0.7π. The suppression effect due to increasing the polarization angle can also be found in the current. Heat generation varies with polarization angle periodically with the period 2π. Heat generation increases with increasing magnitudes of temperature. Our results provide new insight into the heat generation manipulation in spintronic nano-devices, which will be helpful in solving the thermal dissipation problem of high-density integrated circuit with spintronic nano-devices in the future.
